# Focus groups revealed how community health workers in North Carolina find, verify, and process health information for migrant and seasonal farmworkers

**DOI:** 10.1111/hir.12445

**Published:** 2022-06-19

**Authors:** Catherine E. LePrevost, Leslie E. Cofie, Jamie E. Bloss, Joseph G. L. Lee

**Affiliations:** 1 Department of Applied Ecology, North Carolina State University, Raleigh, North Carolina, USA; 2 Department of Health Education and Promotion, College of Health and Human Performance, East Carolina University, Greenville, North Carolina, USA; 3 Laupus Health Sciences Library, East Carolina University, Greenville, North Carolina, USA

**Keywords:** health education, health information needs, health professionals, information dissemination, information literacy, information seeking behaviour, patient education, research, qualitative

## Abstract

**Background:**

Community health workers (CHWs) bridge the gap in health and social services delivery for marginalized communities, providing critical health information to those with limited access to health resources.

**Objectives:**

The purpose of our study was to understand CHWs' approaches to identifying salient and credible health information for migrant and seasonal farmworkers in rural North Carolina (NC).

**Methods:**

Two focus group discussions were held with CHWs in eastern NC and one in western NC in February 2020.

**Results:**

CHWs seek health information on chronic health conditions disproportionately experienced by farmworkers such as diabetes and high blood pressure. They search for information from existing resources in their possession, via the internet, and through consultation with health professionals. CHWs also verify the information and transform the content into resources that are accessible to farmworkers.

**Discussion:**

Our findings suggest that CHWs possess a strong set of information literacy skills that could be enhanced through additional training in crediting sources, creating new materials, and organization and storage.

**Conclusion:**

This study adds to the very limited body of knowledge about how CHWs seek and transmit information to their communities and sheds light on their information need and literacy abilities.

## BACKGROUND

Community health workers (CHWs) are an integral part of health care systems around the world, helping build relationships between medical professionals and their communities (Rhodes et al., 2017). CHWs link communities and health systems across culture and language; a core role for CHWs is providing culturally appropriate health education and information and a core competency for CHWs is maintaining a knowledge base (Community Health Worker Core Consensus Project, 2018). CHWs are thus uniquely positioned to bridge the gap in health and social services delivery for marginalized communities, and they provide critical health information to many communities with limited access to health-related resources. One such marginalized community served by CHWs is the migrant and seasonal farmworker population in the United States (US). According to the National Agricultural Workers Survey, farmworkers predominantly identify as Latinx (83%), report being born in Mexico (69%) and speak Spanish (77%) (Hernandez & Gabbard, 2018). Their average level of formal education is eighth grade (Hernandez & Gabbard, 2018), and they typically live in rural, geographically isolated areas (Keim-Malpass et al., 2015).

CHWs help farmworkers overcome barriers related to language, education and transportation to access health services and information. CHWs have an important distinction from other health workers in that they typically are from the community they serve and usually do not have formal training in a clinical health profession (Pérez & Martinez, 2008). CHWs emerged as a profession in 1920s China and by the mid-twentieth century were common in Latin America as *promotores de salud* (Perry et al., 2014). In the US, the federal government first formally funded CHWs in the Federal Migrant Health Act of 1962 (Pérez & Martinez, 2008). In North Carolina (NC), CHWs have a long history in farmworker health where they identify farmworker housing locations, link farmworkers to migrant health centres, assist with transportation, provide interpretation, work to help health centres provide culturally appropriate care and case management, and provide health education (Lambar & Thomas, 2019). Approximately 60 CHWs work across NC each season providing these services (Harwell et al., 2022).

Despite the important role that CHWs assume in farmworker communities, there is a dearth of literature on their health information-seeking behaviours and the ways that they transmit information to farmworkers (Bloss et al., 2021). Scant previous studies of CHWs serving other populations include a cross-sectional study in Northern India that reported how CHWs accessed materials, their preferences for materials and health information topics for which they needed more information (Raj et al., 2015). In the US, a study interviewed CHWs working for a rural health centre and observed these CHWs to learn about their information practices and their use of information technologies in their workplaces in eastern Kentucky (Shapiro, 2020). Both Shapiro (2020) and Raj et al. (2015) called for more qualitative studies that include CHW voices in different settings and information environments.

In the absence of literature on CHWs' information-seeking behaviours, particularly among CHWs serving farmworkers, research examining health information-seeking among the US Latinx community suggests a variation in access to and use of health information resources and related technologies. Gonzalez et al. (2019) found that foreign-born Latinos of all ethnic subgroups had lower rates of online health information-seeking behaviour. Additionally, Gonzalez et al. (2019) found that US-born individuals within the Mexican subethnicity were generally more likely to engage in health information-seeking behaviour or utilize online patient portals than their foreign-born counterparts, with differences by sub-population, and the authors attributed these disparities in health information-seeking behaviours, in part, to access to both the internet and to health care. In their investigation of low-socioeconomic status Latinx adults' use of smartphones, Kim and Zhang (2015) highlighted the importance of public Wi-Fi for this population to access health information as well as the need to improve Latinx adults' smartphone literacy and health literacy. Taken together, findings from limited prior research on health information-seeking among the US Latinx community reveal a need for improved internet access.

## OBJECTIVE

Knowledge of how CHWs access and utilize health information, though often overlooked in the literature, offers novel insights into promoting the wellbeing of marginalized farmworker communities. Thus, in this study we explored CHWs' approaches to identifying salient and credible health information for migrant and seasonal farmworkers in rural NC, including their various methods of searching for information, organizing information and finally transforming information for effective delivery to farmworkers. The overarching goal of the project is to increase knowledge and access to health education resources among both farmworkers and the CHWs who engage with farmworkers.

## METHODS

As part of a National Library of Medicine-funded project to address health disparities experienced by migrant and seasonal farmworkers, we conducted three focus groups discussions (FGDs) in February 2020 with CHWs in NC to explore CHWs' information-seeking behaviours, as well as their experiences providing health education to farmworkers and their technology needs. The overall project and specific FGD protocols were approved by East Carolina University and Medical Center Institutional Review Board (#19–001817). FGD participants were compensated with $25 gift cards.

### Setting

Two FGDs were held in central/eastern NC and one in western NC. The FGDs were conducted at county Cooperative Extension Centres that were geographically central.

### Participants

Participants were currently employed by outreach or community health organizations that serve migrant and/or seasonal farmworker populations in NC. We invited CHWs to participate in FGDs through announcements on CHW organization listservs and at their events, as well as through direct phone calls and emails. Using convenience sampling, we recruited a total of 28 CHWs.

### Data collection

A semi-structured focus group guide was prepared in English and professionally translated into Spanish. It contained three main sections: preparation for health education outreach, delivery of health education and technology related to health outreach. A native Spanish speaker fluent in English who has facilitation experience used the guide to facilitate the 60-min FGDs. Two FGDs were conducted in Spanish and one in English. The FGDs were audio recorded and later transcribed by the facilitator into English. The facilitator and project team members participated in debriefing sessions after each FGD and after reviewing the transcripts from all three FGDs. The number of FGDs was sufficient to draw conclusions and achieve saturation of themes (Carlsen & Glenton, 2011; Guest et al., 2006).

### Positionality statement

Our research team is led by a health behaviour researcher with extensive experience working with the farmworker community and organizations addressing the needs of this community, an Agromedicine extension specialist with a longstanding relationship with CHWs in NC, a health sciences librarian with expertise in health information literacy development and training, and an evaluator with training as a qualitative research methodologist. As a multidisciplinary team we leveraged the expertise of our advisory board, which included a health education specialist, librarians, CHWs and students from farmworker families. The unique experience, training and identities of the study team informed the analysis and interpretation of the study's findings. The advisory board provided input during the study implementation, development of the focus group discussion guide, data collection and analysis and interpretation of the findings.

### Data analysis

The transcripts were analysed with NVivo 10 by research team members. Team members developed a preliminary codebook by consensus, using both deductive codes derived from the focus group guide and inductive codes from a close reading of all transcripts (Gibbs, 2018). In applying the codebook to the transcripts, the team members met regularly to refine the codebook and conduct coding quality checks for coding reliability. Subsequently, thematic code summaries for key codes were developed and analysed for relevant themes by comparing the code outputs, thematic code summaries, and memos maintained by team members throughout the analytics process. This enabled the team to explore CHWs' experiences and processes of searching, evaluating and processing health information that they deliver to farmworkers. Also, the memos served as an audit trail and facilitated data interpretation (Saldaña, 2015).

## RESULTS

The participants of the FGDs self-identified as being of Latinx ethnicity (93%) and female (71%) and reported having completed high school or some college (54%). Most participants were geographically located in eastern NC (79%). We present here three main findings: the farmworker health information CHWs seek, CHWs' strategies for searching for health information, and CHWs' health information processing approaches. We provide illustrative quotations for these themes.

### Farmworker health information CHWs seek

During the FGDs, CHWs described the most important health education topics for farmworkers as being dependent on several contextual factors. As one participant explained, ‘we need to focus on the needs of each worker, because they are not all going to be the same. They each have different needs, so we need to try to find the subject that they are each interested in at the time’ (P3, FGD1). Several participants specified that the most important topics for farmworker education were based on the characteristics of the farming season (e.g., flooding, excessive heat, COVID-19 pandemic); types of farmworkers and their work experience (e.g., ‘recurring workers who are coming every year or… new workers’ (P3, FGD1); CHWs' previous experiences with farmworkers; and observed and reported needs of farmworkers.

Because of the aforementioned dependence on contextual factors, participants identified numerous health topics that were relevant for farmworkers. The most prevalent responses across all FGDs related to chronic health conditions disproportionately experienced by farmworkers (Bloss et al., 2021). These chronic conditions included diabetes, elevated cholesterol and high blood pressure. CHWs also identified occupational issues—such as pesticides, sun, heat, wet feet, backaches and mosquitos—and crop-dependent health effects related to hemp and tobacco sickness. Participants mentioned infectious diseases, specifically flu, cold, coronavirus and sexually transmitted infections, as being important. Individual CHWs described a variety of other topics related to farmworkers ‘being healthy’ (P3, FGD3): sanitation, nutrition, food safety, alcohol sickness, smoking cessation, dental health and behavioural health.

### CHWs' strategies for searching for information

CHWs described three strategies they employ when seeking health information to disseminate to farmworkers. They search for information from existing resources in their possession, via the internet and through consultation with health professionals. Each of these approaches enables CHWs to acquire resources they deem to be relevant, accurate and accessible to farmworkers.

#### Existing resources

Several CHWs explained that they rely on information from their central office and from webinars and other trainings to find health information needed for health education or to answer specific farmworker questions. Participants reported that their central office had provided various resources, including pamphlets, flyers, posters and videos. One FGD participant explained, ‘[T]he information that the central office gives us has been very useful because it’s easy to understand. So, it’s easy to hand out to the workers’ (P5, FGD3). Other participants additionally highlighted the resources derived from attending trainings: ‘We've gotten some [educational resources] from prior trainings that we have gone to that we can see that would be helpful for [farmworkers]. So, we kind of, you know, get those and make copies and add them in the packets that we give out too’ (P8, FGD2). CHWs further explained that they have ways to store this information, including in books and filing cabinets. One participant elaborated:

… *[W]e're always looking for new things. So, we typically gather information well before season and have it prepared. We have a book that we keep a lot of our education information in*…*. [W]e take that information and we put it in a notebook and then we have a filing cabinet that we also keep information in*. (P1, FGD2)

This participant, along with others, emphasized the need to constantly add new materials to their health education and information repositories. Therefore, having a system to ensure that the information is easily accessible enables them to regularly update and prepare relevant health information for farmworkers.

#### Internet

Participants described the internet as a ‘basic’ way to find information (P1, FGD3), allowing them to access a broader range of resources than they find in their existing repositories. One participant explained, ‘We look for anything that we dont’ have on hand online, like flyers or something like that, that we can print and make copies. We put them together in our box’ (P7, FGD1). Several participants described searching online for health information in response to a particular farmworker or seasonal need: ‘We do [team] meetings…and then we see what the biggest need was last year in the agricultural community…. We go to the internet, and if there is something we dont’ know we try to help each other’ (P8, FGD1). Participants reported using the internet to look for information about emerging health topics, including the coronavirus and health effects associated with working in hemp production.

In searching for online resources, participants reported sifting through available online results. Search results often presented a range of information that required careful review from participants. They described their process for reviewing the information, which included identifying ads that may represent irrelevant or unreliable sources, reviewing and evaluating multiple sources to verify the trustworthiness of the health information, and seeking input from their organization or supervisor to confirm the accuracy of the information they plan to use. One participant (P1, FGD2) offered the following insight about her process for searching for information online:

…I try to pay attention to ads and try to narrow it down because I want to know more information about it. So, I sometimes end up reading four articles and still something else comes out, so I still keep on digging in and digging in more information until I receive an email or something that either my supervisor or another person in the institution sends me and sends me a link like, ‘This is what we need to do for patients.’ So that’s when I [say], ‘Okay, this is kind of almost the same [as what I found].’

The internet also improves CHW access to specialized health information through training and webinars: ‘Basically, it’s the computer and the internet. We are very dependent as far as training goes. Many times, we cant’ go to a specific training, but there may be a very good webinar offered, so we can see everything that we need there, at the office’ (P2, FGD1). Some CHWs, however, emphasized that while they rely on the internet, information that is specific to farmworkers is not always available: ‘The internet has everything. There is a lot of information as far as general health is concerned, but we also have to be certain the information is fine to give it to the people that we see, to the farmworkers. So the information has to be more focused on their health, such as about backaches, pesticides, and so on’ (P5, FGD3). Participants expressed an awareness of both the affordances and limitations of the internet as a means of searching for farmworker health information.

#### Health experts

As part of their process of identifying and confirming health information, participants explained that they seek out the expertise of specific individuals—through ‘the central office,’ ‘someone with experience working with farmworkers,’ a ‘doctor who we're usually in touch with,’ and ‘clinics or hospital…from the people who have experience’. They also described contacting related state agencies and national organizations: ‘Sometimes I gather information from partners that operate nationwide, like Health Outreach Partners, like Migrant Clinicians Network, or MHP Salud; they gather information according to how they see that need is during that year so they provide modules or brochures that are pertinent to what we do in outreach’ (P2, FGD2). CHWs described seeking out these expert sources for training, educational resources and information outside of CHWs' expertise. One participant explained, ‘If there is something for which we dont’ have enough information, we have a lot of contacts [who are] doctors and nurses, so they become sources of education’ (P5, FGD1). In addition to experts' sharing reliable resources and providing answers to questions CHWs cannot answer, CHWs also described relying on experts to confirm or approve information CHWs find in their searches for farmworker health resources.

### CHWs' health information processing approaches

Once they identify relevant health information and related health resources for farmworkers, CHWs explained that they verify the information and transform the content into resources that are comprehensive and accessible to farmworkers. Some CHWs noted the dangers of overlooking the source and credibility of the information they find. Hence, several participants expressed that, in their work to provide useful information to farmworkers, they are acutely aware of the limits of their expertise and have ways of verifying the information to avoid potential misinformation that could harm farmworkers.

#### Verifying online information

While participants universally recognized the value of the internet, some raised concerns about the reliability of the information found through online searches. One participant (P3, FGD3) expressed particular apprehension about the trustworthiness of information acquired through online searches: ‘I have to say, sometimes I'm afraid of the internet, because you can find many things, and some things are more trustworthy. I, in fact, do have some reservation. I'm not saying I dont’ use it, but you do have to look a lot to see what you find and what it is that you'll inform [farmworkers], because it can be dangerous. It can be very useful, but at the same time it can be very dangerous’. In response to these concerns, CHWs reported employing two approaches to verify online information—using sites they perceive to be reliable and confirming information found online with someone at their organization. Participants indicated that ‘websites that are validated by the government, like the CDC’ and ‘university websites’ (P2, FGD1) are ‘sources of information that are well known’ and ‘valid’ (P9, FGD1). Other CHWs specified health departments and state and federal agencies as having reliable websites. CHWs further suggested that their search for reliable content is based on the ‘standards, policies and procedures of their organization’ (P1, FGD2). Specifically, one (P1, FGD2) explained: ‘we look at…what our providers are using as far as different topics, especially hypertension, blood pressure, what their guidelines are and some of the websites that they pull actually for their education that they give their patients in the clinic’.

As one participant (P4, FGD2) described, CHWs often follow up their web searches by seeking verification and approval from their organizations:

I think for us, like a lot of information we get from providers as well and, like, our clinic, but if we have something new, for example like the coronavirus, we try to use websites that we know that you can actually trust. That information and obviously confirm that with our program to make sure that it’s okay to share this information. We all agree that this is like the newest information that’s out there.

Participants reported that their supervisors, physicians and communication specialists suggest websites for CHWs to search, send CHWs preferred resources, and ensure that information that CHWs find is accurate and in the correct format to share with farmworkers.

#### Reformulating information

Participants described engaging in processes to make health information more understandable and appropriate—linguistically, culturally and educationally—for farmworkers. They reported that after obtaining health information (e.g., from their central office and the internet) they are responsible for transforming the information into easily accessible educational materials. For example, one CHW (P3, FGD3) explained, ‘[O]ur homework is to put it in language that the patient understands, because we have educational barriers and barriers to medical expression that can make it difficult for a patient to understand’. To avoid providing ‘information that is too advanced’ or ‘overload[ing] the workers,’ CHWs explained that they ‘pull key things [information] out’ of health resources and prepare handouts with ‘key little things that [they] want [workers] to remember’ (P1, FGD2). Participants further elaborated that they adapt resources to fit within a specific time frame and educational context:

So we have to think, ‘What can we bring that has quality in 15 minutes?’… So we are focusing right now in working more in popular education, by adapting our resources to different times and different learnings. We create our own material as well; we try to make it so that it is very educational. We look for interactive tools with the worker, so that we can engage them in our health education. It can be from five minutes to an hour. Even when we are driving a patient from their home to the clinic, we take advantage of that moment to educate. (P2, FGD1)

In addition to highlighting how CHWs extract the most salient information to share with farmworkers and make information more meaningful to farmworkers specifically, this quotation reflects how CHWs consider the delivery format of health education to make it more engaging.

#### Limitations to CHWs' roles

CHWs revealed that they conceptualize their roles as being limited to providing health education. They demonstrated a keen level of awareness of the scope of their work and expertise. As explained by a CHW, ‘We know we are health educators, but we also know that the moment comes when we have to refer them to a medical appointment’. Participants were well-informed about where to refer farmworkers to find information that CHWs are not comfortable with providing. They also understood how to access information that they may not have readily available while in the field. One CHW explained:

And sometimes whenever we dont’ know the answer to whatever they're asking, we just let them know, ‘I dont’ know much about it. I dont’ want to give you the wrong information, but let me go ahead and go back to the office, try to gather as much information as possible.’ And then come back to the second visit and provide them with that information that he's needing. (P2, FGD2)

CHWs all described the limits of their health expertise and, as explained by most participants, were intentional in their efforts to leverage their health information resources and networks to address farmworkers' health related concerns.

## DISCUSSION

### Principal findings

The purpose of our study was to understand CHWs' approaches to identifying salient and credible health information for migrant and seasonal farmworkers in rural NC. We identified three main findings, including the types of farmworker health information sought by CHWs, their strategies for searching for information, and subsequently their methods of processing the information for effective delivery to farmworkers. CHWs indicated that they utilize their own existing resources, the internet, and consultation with health professionals in their search for accurate and relevant health information for farmworkers. To ensure that the information is accurate, CHWs reported that they verify the information, including by using trusted sources from the government, universities and their own internal organizations. They also modify the content and format of the information when needed to make health information accessible to farmworkers. Further, in light of the scope and importance of their outreach to farmworkers, CHWs were cognizant of the limits of their roles as health educators and thus were intentional about providing information within their expertise.

Given there are few prior studies on the health information-seeking behaviours of CHWs, our findings improve the understanding of CHWs' approaches to identifying health information, specifically to promote the wellbeing of marginalized farmworker communities. In comparison to the CHWs in Raj et al.'s (2015) assessment of information and communication technologies and training in India, the CHWs in our study described having increased access to technology and resources and demonstrated greater information literacy skills. Our findings resonated with those of Shapiro (2020) in that CHWs in both studies helped process and deliver information to their communities, sought information from the internet and leveraged their networks.

### Findings in context

While the health information topics that CHWs deemed most important were highly dependent upon contextual factors, the most identified topics were chronic health conditions experienced by farmworkers, followed by occupational hazards and related health conditions. In a recent mapping review of the literature (Bloss et al., 2021), the authors found an existing scientific literature on chronic conditions such as diabetes (*n* = 13 papers specific to farmworkers) and a literature on occupational hazards (e.g., *n* = 66 papers on injury prevention and *n* = 130 papers on pesticides). However, the authors found just 89 of the 1083 identified papers on farmworker health to be describing or evaluating health promotion efforts or interventions. Thus, while CHWs may face barriers to accessing scientific literature, there are also likely gaps in the available evidence that could be used to inform their work.

**FIGURE 1:**
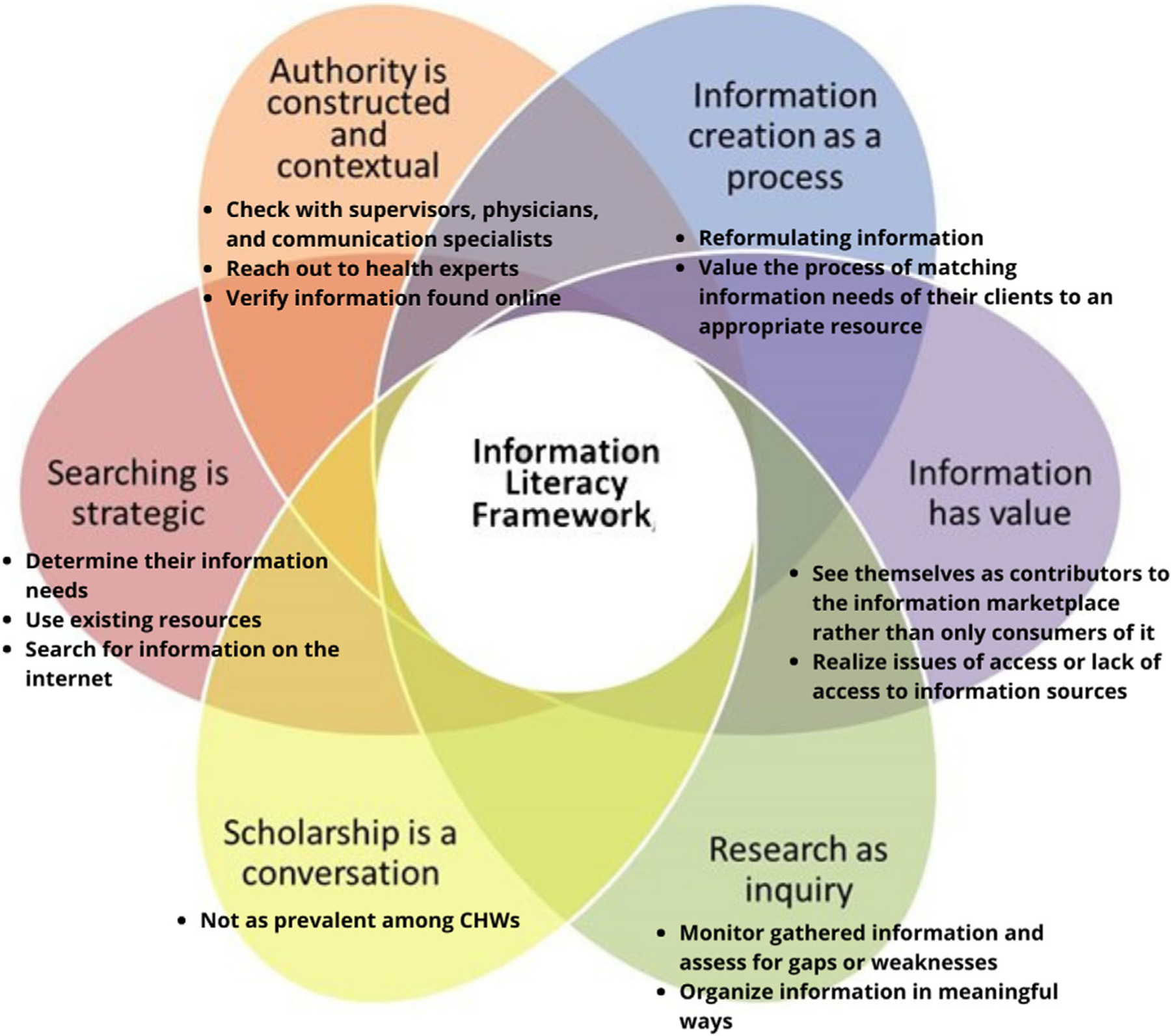
Alignment of information behaviours of farmworker-serving CHWs in NC with the ACRL framework for information literacy.

Given CHWs' core role of providing health education and information as well as their core competency of maintaining a knowledge base (Community Health Worker Core Consensus Project, 2018), CHWs' work is informed by information literacy tenets. Future work on CHWs' engagement with the scientific literature and their information-seeking behaviours could consider the framework for information literacy prepared by the Association of College & Research Libraries (ACRL, 2016). The ACRL’s *Framework for Information Literacy for Higher Education*, which has six tenets, is intended to allow librarians and researchers to assess information literacy skills and design instructional materials according to learner needs. As shown in Figure 1, participants exhibited skills and information-seeking behaviours representing many of the framework's tenets. CHWs' description of their search for health information to address gaps in their knowledge and education resources demonstrates their understanding of two central tenets: ‘searching as strategic exploration’ and ‘research as inquiry’. Many participants indicated that they gathered information based on the needs of farmworkers and subsequently monitored that information to determine its quality. They also reported using filing systems to organize the information for easy access.

CHWs demonstrated their understanding that ‘authority is constructed and contextual’ by verifying information found online and expressing scepticism of information sources. They also reported checking with supervisors, physicians and communication specialists who suggest reliable sources; thus, they are actively defining different types of authority and using research tools and indicators of authority to determine credibility of sources. Participants revealed their recognition of the concept of ‘information creation as a process’; CHWs described assessing if an information product meets a particular information need and reformulating information when necessary. The final two tenets in the framework, ‘information has value’ and ‘scholarship as conversation’, were less prevalent in our discussions with CHWs. Participants did not discuss citing sources or crediting original creators. However, in the context of ‘information has value’, participants did present themselves as contributors to the information marketplace by saving, storing and repackaging information for dissemination to farmworkers. In doing so, participants revealed their recognition of issues of access—specifically lack of access—of information sources that are appropriate for farmworkers. The absence of the ‘scholarship as conversation’ tenet may not be surprising given that CHWs operate outside of an academic setting and are likely unfamiliar with the process of publishing scholarly products.

Although we were able to link participants' responses to the tenets described above through this qualitative research study, the findings are self-reported in terms of how the participants in the focus groups described their information seeking processes and skillsets related to delivering health education. Our findings do not reflect an objective assessment of the participants' information literacy competencies, and future studies measuring CHWs' ability to find and interpret health information would allow for comparison with participants' descriptions.

Overall, participants demonstrated knowledge of many of the tenets included in the ACRL framework. Interpreting our findings through the lens of the ACRL framework suggests that future training for CHWs should focus on the tenet ‘searching as strategic exploration’ to improve CHWs' ability to access a variety of information resources, including library databases. Furthermore, enhancing CHWs' advanced search skills (e.g., advanced Google searches) would improve efficiency and the quality of resources identified. Other areas for professional development for CHWs, informed by the tenets ‘scholarship as conversation’ and ‘information has value’, include citing sources and crediting original creators, creating new patient education materials and designing materials at appropriate literacy levels, and strategies for the organization and storage of educational materials. Librarians and public health or medical professionals should partner to support these endeavours. We have described elsewhere our team's efforts to create resource lists, videos and webinars to support the professional development of farmworker-serving CHWs in NC (Bloss et al., 2022).

### Limitations

Whereas the present study provided information on the experiences of CHWs in searching for and accessing health information for farmworkers, there are a few limitations worth noting. The findings may not be generalizable to CHWs in other states and working with other marginalized populations, as this study was conducted solely in the state of NC with CHWs who work with migrant and seasonal farmworkers. Additionally, participants self-selected for focus group participation, and CHWs who declined our invitations may have different perspectives. Strengths of the research include the high number of participants relative to the total number of farmworker-serving CHWs in NC, rigorous qualitative methodology, use of both English- and Spanish-language focus groups, and data collection across central/eastern and western NC.

## CONCLUSION

This study adds to the very limited body of knowledge about how CHWs seek and transmit information to their communities, and sheds light on their information needs and information literacy skillsets. Additional research on patterns in CHWs' information-seeking behaviours should be conducted in other parts of the US and internationally to determine if our findings related to CHWs' information-seeking behaviours are consistent with other CHWs' based on their populations served and geographic locations. An improved understanding of CHWs' information needs can inform professional development activities to support the work of CHWs that serve as health intermediaries to vulnerable populations like farmworkers. As information literacy (i.e., information retrieval and transformation) is a skillset needed to be successful as a CHW, future trainings or programs should be developed that focus on teaching CHWs how to credit sources; create new, tailored patient education materials; and how to best organize and store health education materials. Librarians can serve as key partners with public health or medical professionals in this effort to offer more training to CHWs and further the proposed research.

## Key Points

1. As information literacy is a necessary skillset to be successful as a community health worker, training for community health workers should include this skill development.
2. Opportunities for community health worker training include crediting sources, creating new materials, and organization and storage.
3. Librarians and public health or medical professionals should partner to support efforts to train community health workers.
4. Patterns in community health workers' information-seeking behaviours in the United States and internationally should be examined according to populations served and geographic location.
